# High vs. low tidal volume and pulmonary complications in patients with cervical spinal cord injury on mechanical ventilation: systematic review

**DOI:** 10.3389/fmed.2024.1362318

**Published:** 2024-03-01

**Authors:** Edinson Dante Meregildo-Rodríguez, Gustavo Adolfo Vásquez-Tirado, Claudia Vanessa Quispe-Castañeda, María del Carmen Cuadra-Campos, Jhuliana M. Contreras-Cabrera, Juan Luis Pinedo-Portilla

**Affiliations:** ^1^Escuela de Medicina, Universidad César Vallejo, Trujillo, Peru; ^2^Facultad de Medicina Humana, Universidad Privada Antenor Orrego, Trujillo, Peru; ^3^Servicio Cuidados Intermedios, Hospital Nacional Almanzor Aguinaga-Asenjo, Lambayeque, Perú

**Keywords:** cervical spinal cord injury, tidal volume, mechanical ventilation, ventilator associated pneumonia, systematic review, meta-analysis

## Abstract

**Introduction:**

Cervical spinal cord injury (CSCI) patients on mechanical ventilation often lack standardized guidelines for optimal ventilatory support. This study reviews existing literature to compare outcomes between high tidal volume (HTV) and low tidal volume (LTV) strategies in this unique patient population.

**Methods:**

We searched for studies published up to August 30, 2023, in five databases, following a PECO/PICO strategy. We found six studies for quantitative analysis and meta-analyzed five studies.

**Results:**

This meta-analysis included 396 patients with CSCI and mechanical ventilation (MV), 119 patients treated with high tidal volume (HTV), and 277 with low tidal volume (LTV). This first meta-analysis incorporates the few studies that show contradictory findings. Our meta-analysis shows that there is no significant statistical difference in developing VAP between both comparison groups (HTV vs. LTV) (OR 0.46; 95% CI 0.13 to 1.66; *p* > 0.05; *I*^2^: 0%), nor are there differences between the presence of other pulmonary complications when treating with HTV such as acute respiratory distress syndrome (ARDS), atelectasis, onset of weaning.

**Conclusion:**

In patients with CSCI in MV, the use of HTV does not carry a greater risk of pneumonia compared to LTV; in turn, it is shown as a safe ventilatory strategy as it does not establish an increase in other pulmonary complications such as ARDS, atelectasis, the onset of weaning nor others associated with volutrauma. It is necessary to evaluate the role of HTV ventilation in this group of patients in primary RCT-type studies.

## 1 Introduction

Cervical spinal cord injury (CSCI) usually entails the need for constant ventilatory support as mechanical ventilator (MV), requiring mechanical ventilation immediately after the injury in most cases (1–3).

The management of ventilatory support in CSCI patients is not standardized according to their specific needs, since existing management protocols based on multiple clinical trials for optimal mechanical ventilation settings are designed for patients with acute respiratory distress syndrome (ARDS) without neurological injury (4–7). In these protocols it is suggested that an optimal tidal volume (TV) is 4 to 6 ml or 6 to 8 ml, since this range is considered safe due to the lower incidence of atelectasis, barotrauma and mortality (8, 9). However, there is a lack of research regarding optimal ventilator settings in people with acute CSCI.

Currently guidelines on acute spinal cord injury recommend high tidal volume (HTV) up to of >15 ml/kg predicted body weight (10). Peterson et al. (4) carried an investigation in patients with CSCI connected to MV and observed that patients managed with high tidal volumes had a lower frequency of ventilator-associated pneumonia (VAP), shorter duration of weaning time, and lower incidence of atelectasis compared to a low tidal volume group (LTV) (4). Other studies performed in CSCI populations reported that HTV management was not associated with major complications, suggesting that it is safe to use (5, 7, 11–13).

In CSCI patients with HTV exposure, the maximum values of airway pressure with higher volumes than the standard do not usually exceed 30 cm H_2_O due to the flaccidity of muscle tone in these patients, representing a potential benefit (14, 15). On the contrary, LTV fail to compensate for profound muscle weakness, and is associated with an increased need for sedation, mucosal obstruction, decreased surfactant production and increased incidence of atelectasis (16–21). It has even been reported that in quadriplegic patients a lower TV translates into greater dyspnea (11).

Due to the lack of consensus and a high level evidence on adequate ventilatory support settings in the CSCI population, we performed a systematic review and meta-analysis to revisit the recommendations that are being widely followed and provide data that will support decision making in regards to the respiratory support.

## 2 Materials and methods

### 2.1 Search strategy

Our systematic review was performed following the guidelines outlined in the Cochrane Handbook for Systematic Reviews (22), PRISMA (23), and AMSTAR 2 (24). The protocol was preregistered in PROSPERO (CRD42023452844). Thorough searches were conducted across multiple databases, including MEDLINE (PubMed), Scopus, EMBASE, Web of Science, Science Direct and the Cochrane Library. Database screening involved the application of thesaurus (MeSH, Emtree, etc.), free terms, and their synonyms. Using boolean operators, we implemented our PECO/PICO strategy (Population: adult patients with cervical spinal cord injury in mechanical ventilation; Exposure: ventilation with high tidal volume; Comparator: ventilation with normal tidal volume; Outcome: ventilator-associated pneumonia OR intrahospital mortality OR total weaning days OR other pulmonary complications). Keywords included terms related to exposure, such as “cervical spinal cord injury” OR “cervical spinal cord trauma” OR “tidal volume,” and outcome-related terms, such as “ventilator-associated pneumonia” OR “pulmonary complications.” The detailed search strategy is available in the (Supplementary Table 1A).

**FIGURE 1:**
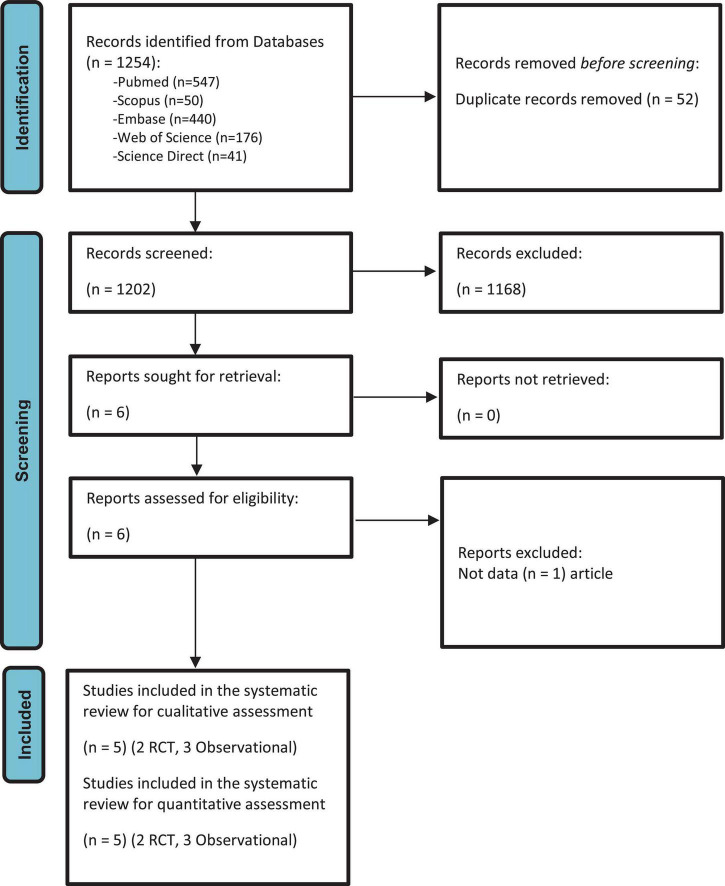
PRISMA 2020 flow diagram of the selection process of the primary studies included.

All the articles identified through primary and secondary screenings were compiled using Zotero^®^ 6.0.15. Following the duplicate removal, the documents were stored in the Rayyan^®^ tool, where two authors (EDMR and MCCC) conducted individual screenings of titles and abstracts independently (blinded). The selection of studies was achieved through consensus, and in instances of disagreement, a third researcher served as the arbitrator (GAVT). The chosen papers underwent a second full-text review to assess eligibility. A secondary manual search of reference lists and citing articles of included publications were also reviewed to increase the identification of relevant studies. The selection process is explained in detail in Figure 1.

### 2.2 Selection criteria

We included observational studies (RCS) and randomized controlled trials (RCTs) that included adult patients (≥18) diagnosed with CSCI in need of MV assistance for more than 2 weeks and less than 6 months, with cervical lesions classified as AIS A, B, or C and initiated without pre-existing pulmonary pathology. We included articles published up until August 30, 2023, with no restrictions on date or language. Case reports, case series, and duplicated publications were excluded.

### 2.3 Outcomes

The primary outcome the frequency of VAP, defined as the occurrence or progression of new pulmonary infiltrates with at least two of three signs: temperature >38 or <36, leukocytosis or leukopenia, or left deviation of immature forms (10%), along with tracheobronchial purulent discharge (11, 12). Secondary outcomes included the presence of acute respiratory distress syndrome (ARDS), atelectasis, and composite mortality (VAP and overall mortality).

### 2.4 Data extraction

Two independent researchers, blindly, gathered and extracted relevant details of each included study using and standardized spreadsheet, including authors names, country and year of publication, clinical and epidemiological characteristics of the population, number of participants and cases, measures of association, confounding factors, and the outcomes. For dichotomous and time-to-event variables, we compiled odds ratios (OR), risk ratios (RR) and hazard ratios (HR) with 95% confidence intervals (CI 95%). Missing data were reported when appropriate.

### 2.5 Statistical analysis

We used the Mantel-Haenszel method in the meta-analysis to pool adjusted ORs with 95% CIs. All studies reported pooled ORs, none RRs. We conducted this meta-analysis using R^®^ 4.2.226 software. To summarize the quantitative synthesis, we used forest plots with the library meta, function metabin, and Mantel-Haenszel method with Restricted Maximum Likelihood (REML) for tau (2). Our protocol stated that we would examine heterogeneity among studies with Cochran’s *Q*-test and Higgins *I*^2^ statistic. If heterogeneity was not statistically significant (*p* > 0.10, *I*^2^ statistics <40%), we would use a fixed effects model. On the other hand, we would use a random effects model if heterogeneity was statistically significant (22). We conducted sensitivity a using the function InfluenceAnalysis. Subgroup analysis could not be performed due to the small number of patients in the studies collected.

### 2.6 Quality assessment

We assessed the potential risk of bias using both the Risk Of Bias In Non-randomized Studies–of Exposure (ROBINS-E) (25) and the Cochrane risk-of-bias tool for randomized trials (RoB 2) (26). To examine the possibility of publication bias, we employed a funnel plot and Egger’s test calculation (27).

Two researchers (EDMR and MCCC) assessed the certainty of the evidence (CoE) of the study outcomes for each outcome based on the Grading of Recommendations Assessment, Development, and Evaluation (GRADE) criteria (28, 29). Any discrepancies between the reviewers were resolved through discussion with the leading researcher (GAVT).

## 3 Results

### 3.1 Search results and study characteristics

Six records were included (4, 5, 7, 11, 12, 30) in the qualitative synthesis in our review (Table 1). Afterward, five articles remained for the meta-analysis (Supplementary Table 1B), three observational studies (4, 7, 10) and two RCTs (11, 12) (Figure 1); with a total of 396 MV patients enrolled, 119 patients with HTV, and 277 with LTV. Among the studies included for qualitative analysis, five were conducted in the USA (4, 5, 7, 11, 30) and one in India (12).

**TABLE 1:** General characteristics of included studies.

References, country	Design	Participants	Exposition	Outcome	Adjustment factors	OR / RR / HR (95% CI)
Peterson et al. (4), EEUU	RCS	Patients with complete tetraplegia for injury at the C3-C4 level, need for 24 h ventilatory support at admission, and successful weaning at discharge between 1983 and 1993. *N* = 42 patients: 19 with high tidal volume ventilation (HTV) and 23 with low tidal volume (LTV). The mean age of the HTV group was 31 years, and of the LTV group was 29 years. Of the total number of patients, 5 were women.	Mechanically ventilated patients who at 2 weeks after admission had an inspiratory tidal volume greater than 20 ml/kg (mean = 25.3 ml/kg; range = 20.3 ± 32.2 ml/kg) vs. patients with inspiratory tidal volume less than 20 ml/kg (mean = 15.5 ml/kg; range = 11.6 ± 19.4 ml/kg).	Successful weaning, duration of mechanical ventilation, pneumonia.	None	No measures of association are reported
Wong et al. (5), EEUU	RCS	Patients with acute tetraplegia due to upper cervical spinal cord injury (C1–C4) admitted 2 years prior to the start of the study. N: 24. Of which 22 were males and 2 females. Mean age was 33.4 years (SD: 16.6). Patients of different ethnicities [African-American (N: 3), Asian (N: 3), Hispanic (N: 7) and white (N: 11)], and etiologies (gunshot wound, motor vehicle accident, fall, diving accident, cervicomedullary tumor, bicycle accident, sports accident) were included. Body mass index had a mean of 25.82 (SD: 16.6).	Quadriplegic patients who received respiratory support with MV according to the protocols of the center to which they were admitted (tidal volume from 12 ml/kg ideal body volume, high-frequency percussive ventilation, mechanical insufflation and exsufflation) vs. baseline respiratory status at the time of admission to the center. Consider HTV at 20 ml/kg vs. standard volumes 8–10 ml/kg	AIS impairment classification, incidents of pneumonia, MV disconnection attempts, types of intervention provided in ventilatory support, patient respiratory findings.	None	No measures of association are reported
Fenton et al. (11), EEUU	RCT	Patients older than 18 years with subacute traumatic tetraplegia less than 6 months, C3–C6 level injuries, non-functional motor preservation as assessed by the AIS scale and requiring continuous mechanical ventilation were randomized to the standard or high tidal volume group. *N* = 33. Patients with diaphragmatic paralysis were excluded	All patients were initially ventilated at 10 ml/kg ideal weight with a PEEP of 5 cm H_2_O for 72 h and then randomized to ventilation with standard tidal volume (10 ml/kg PEEP) or high tidal volume (20 ml/kg PEEP). The use of PEEP is the standard of care at this center and was maintained at 5 cm in both groups.	Safety of exposure to high tidal volumes, weaning time, incidence of pulmonary events (pneumonia, barotrauma, ARDS).	None	There was no significant difference in the number of days to weaning between the two treatment groups. The odds of adverse pulmonary events did not differ between the two groups, and the odds of developing VAP did not differ between the two groups. OR = 1.56 (*p* = 0.1597).
Korupolu et al. (7), EEUU	RCS	Patients with spinal cord injury requiring mechanical ventilation with tracheostomy admitted between 2015 and 2019. *N* = 140. Patients with injury older than 1 year, ARDS, younger than 18 years, severe dysphagia were excluded.	Patients were divided into two groups according to the maximum VT received calculated as ml/kg PBW, patients who received a volume less than 15 ml/kg were included in the moderate VT group (VTM = 50), while those who received a volume greater than 15 ml/kg were included in the high VT group (HTV = 34).	Incidence of pneumonia, adverse events, time elapsed from admission to weaning.	Age, sex, tidal volume at admission	Incidence of pneumonia in HTV vs. MTV. RR = 4.3 *p* = 0.01; CI 95% 1.5–12, the probability of pulmonary adverse effects in the HTV vs. MTV group. OR = 5.4; CI 95% 1.8–17
Hatton et al. (30), EEUU	RCS	Patients with acute cervical SCI due to trauma older than 16 years admitted to that center between the years 2011 to 2018. *N* = 181. Patients with MV less than 2 days, ARDS and a score less than 5 on the abbreviated head injury scale were excluded. Median age was 47 years.	Patients on MV who received tidal ventilation at high volume (mean tidal volume: 10.8 cc/kg PBW) vs. ventilation at standard volume (mean tidal volume: 7.6 cc/kg PBW). HTV initiates an up-titration protocol for TV from 10 to 20 ml/kg considering this value finally, LTV or standard less than 10 ml/kg	Ventilator-associated pneumonia, days from admission to VAP, days from admission to tracheostomy, ventilatory dependency at discharge, discharge disposition, hospital mortality.	Age, ISS, level of injury, type of trauma, completeness of spinal cord injury, mechanical ventilator dependence on day 30, hospital stay.	Ventilation to HTV is associated with an increased risk of pneumonia. RR = 1.96 95% CI 1.55–2.17, *p* = 0.06 HTV was also associated with increased risk of ventilator dependency. RR = 2.07 95% CI 1.48–2.71 *p* < 0.001.
Sengupta et al. (12), India	RCT	Patients with acute traumatic cervical spinal cord injury admitted to NICU within 24 h of injury with requirement for mechanical ventilation, admitted between September 2018 and April 2019. *N* = 56. Patients with associated head or chest injury, history of aspiration, OSA, body mass index greater than 30 kg/m^2^ were excluded.	Patients were randomly assigned by computerized block system to two groups, high tidal volume group (12–15 ml/kg IBW) and low tidal volume group (6–8 ml/kg IBW).	Successful weaning, barotrauma, atelectasis, VAP, ARDS, duration of treatment, hospital stay, mortality	None	No measures of association are reported

TV, volume tidal; HTV, high tidal volume; LTV, low tidal volume; RCS, retrospective cohort study; RCT, randomized controlled trial; ISS, injury severity score; AIS, abbreviated injury scale; MV, mechanical ventilation; PEEP, positive end-expiratory pressure; ARDS, acute respiratory distress syndrome; VAP, ventilator associated pneumonia; ICU, intensive care unit; NICU, neurotrauma intensive care unit; IBW, ideal body weight; PBW, predicted body weight.

In this investigation, the authors use different definitions about TV, but in general, HTV are considered to be values greater than 15 ml/kg. For example, Peterson: HTV 20 ml/kg, LTV median of 15 ml/kg; Wong: HTV 20 ml/kg, LTV 8–10 ml/kg; Fenton: HTV 20 ml/kg LTV 10 ml/kg; Korupolu: HTV greater than 15 ml/kg LTV less than 15 ml/kg; Hatton: HTV initiates an up-titration protocol for TV from 10 to 20 ml/kg considering this value finally, LTV or standard less than 10 ml/kg; Sengupta HTV up to 15 ml/kg LVT 6–8 ml/kg).

Therefore, TV over 15 ml/kg are considered HTV, and volumes less than this are considered standard or LTV since the controls usually have less than 10 ml/kg except for Peterson (4, 5, 7, 11, 12, 30). The primary outcome across all studies was the presence of VAP, defined as the occurrence or progression of new pulmonary infiltrates with at least two of three signs: temperature >38 or <36, leukocytosis or leukopenia, or left deviation of immature forms (10%), along with tracheobronchial purulent discharge (11, 12). The HTV group developed fewer than 50 cases of VAP, while the LTV group had 130 cases.

Additional demographic characteristics of the study population are documented in Table 1.

### 3.2 Risk of bias in studies

Among the five studies included in our meta-analysis, two were RCTs assesed as “some concerns” risk of bias (11, 12), attributable to the absence of blinding, but no other transgressions were identified in other stages of the study protocol. In contrast, two RCS studies were assesed as “some concerns” risk of bias (7, 30) and one “high” risk of bias (4) (Table 2).

**TABLE 2:** Risk of bias of the included studies.

References, country	Study design	Tool	Conclusion
Peterson et al. (4), EEUU	RCS	ROBINS-E	High risk
Wong et al. (5), EEUU	RCS	ROBINS-E	High risk
Fenton et al. (11), EEUU	RCT	RoB 2	Some concerns
Korupolu et al. (7), EEUU	RCS	ROBINS-E	Some concerns
Hatton et al. (30), EEUU	RCS	ROBINS-E	Some concerns
Sengupta et al. (12), India	RCT	RoB 2	Some concerns

RCS, retrospective cohort study; RCT, randomized controlled trial; ROBINS-E, risk of bias in non-randomized studies–of Exposure; RoB 2, version 2 of the Cochrane tool for assessing risk of bias in randomized trials.

### 3.3 Risk of VAP

**FIGURE 2:**
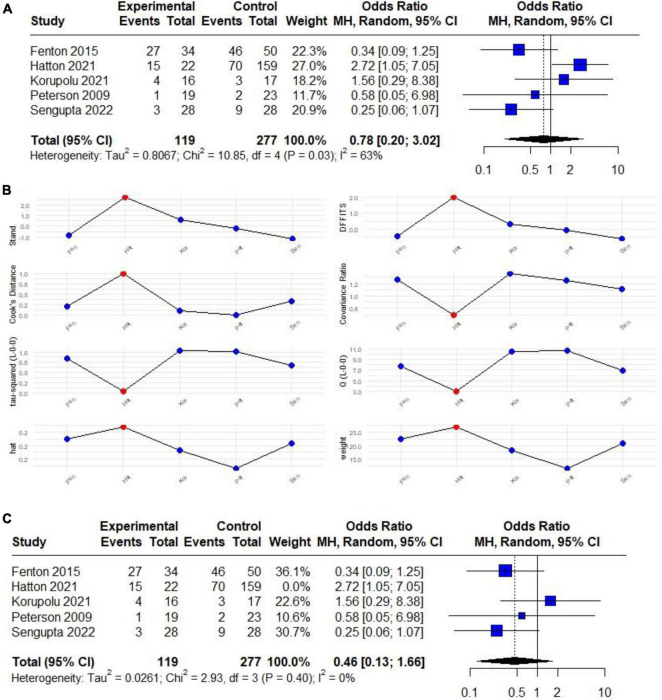
**(A)** Forest plot of the effect of HTV compared with LTV on the risk of developing VAP in patients with CSCI (RCTs and observational) studies. **(B)** Plot of the influence analysis considering (RCTs and observational) studies included in the initial meta-analysis to determine a high heterogeneity, revealing that Hatton behaves like an outlier study. **(C)** Forest plot of the effect of HTV compared with LTV on the risk of developing VAP in patients with CSCI without outlier study.

We conducted an initial meta-analysis including five studies (two RCTs and three observational studies). The analysis revealed that among 119 patients subjected to HTV, 50 exhibited VAP, whereas among 277 patients receiving LTV, 130 developed VAP. The meta-analysis indicated an absence of a significant relationship between the presence of VAP and HTV used (OR 0.78; 95% CI 0.20 to 3.02; *p* > 0.05), with an unacceptably high heterogeneity (*I*^2^: 63%) (Figure 2A).

Due to the limited number of studies, subgroup analysis could not be performed. However, a sensitivity analysis through Influence Analysis revealed that excluding Hatton et al. (30), who behaved as an outlier (Figure 2B), the overall results showed a trend indicating that ventilation with HTV may provide protection against VAP (OR 0.46; 95% CI 0.13 to 1.66; *p* > 0.05) with no heterogeneity (*I*^2^: 0%) (Figure 2C).

### 3.4 Risk of VAP and mortality

**FIGURE 3:**
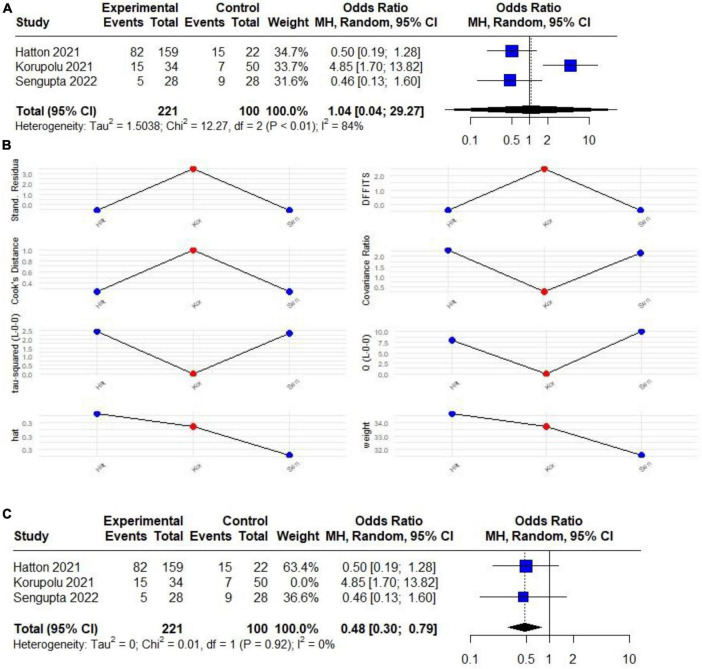
**(A)** Forest plot of the effect of HTV compared with LTV on the risk of developing a composite outcome (VAP and mortality) in patients with CSCI (RCTs and observational) studies. **(B)** Plot of the influence analysis considering (RCTs and observational) studies included in the initial meta-analysis to determine a high heterogeneity, revealing that Korupolu behaves like an outlier study. **(C)** Forest plot of the effect of HTV compared with LTV on the risk of developing a composite outcome (VAP and mortality) in patients with CSCI without outlier study.

Only three studies assessed a composite outcome (VAP and mortality) (7, 12, 30). There was no significant difference in VAP and mortality rates among patients ventilated with both HTV and LTV (OR 1.04; 95% CI 0.04–29.27) with unacceptable high heterogeneity (*I*^2^: 84%) (Figure 3A). Upon further investigation of heterogeneity using Influence Analysis, it was identified that Korupolu et al. (7) acted as a significant outlier (Figure 3B). Upon excluding this, the analysis demonstrated that ventilation with HTV emerged as a protective factor for the composite outcome under consideration (OR 0.48; 95% CI 0.30 to 0.79; *p* < 0.05) with no heterogeneity (*I*^2^: 0%) (Figure 3C).

### 3.5 Risk of other pulmonary complications

When analyzing potential complications associated with HTV, in terms of atelectasis, there is no heightened occurrence in the HTV compared to the LTV group (OR 0.45; 95% CI 0.02 to 9.28; *p* > 0.05), with no heterogeneity detected (*I*^2^: 0%). Similarly, the incidence of complications after tracheostomy did not differ between the HTV and LTV groups (OR 0.45; 95% CI 0.00 to 165.22; *p* > 0.05) without heterogeneity (*I*^2^: 0%). The most recent study by Sengupta et al. (12) exclusively provides data on ARDS showing no significant difference between the HTV and LTV ventilation groups (OR 0.30; 95% CI 0.08 to 1.11; *p* > 0.05).

**FIGURE 4:**
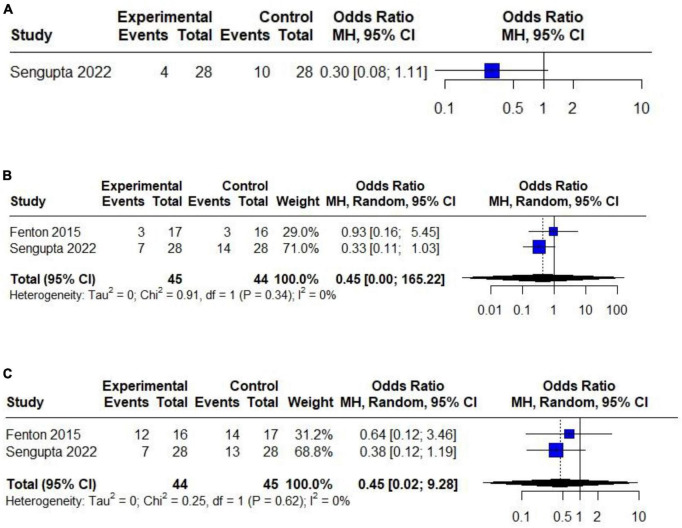
Forest plot of the effect of HTV compared with LTV in patients with CSCI on the risk of developing: **(A)** ARDS (only one RCT). **(B)** Atelectasis (only two RCTs). **(C)** complications after tracheostomy (only two RCTs).

It was not feasible to conduct a meta-analysis for other crucial outcomes, as these data were presented solely in a single study, including parameters such as the time of weaning onset and isolated mortality (Figure 4).

### 3.6 GRADE assessment

**FIGURE 5:**
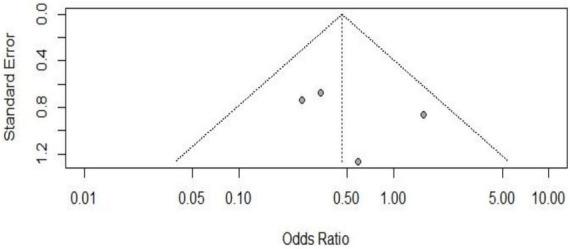
Funnel plot of the included studies in the meta-analysis on the effect of developing VAP in patients with CSCI considering observational and RCTs studies without outlier.

We used GRADE to assess the certainty of evidence (CoE) for the presence of VAP in five studies that involved 396 patients. Despite the small number of studies, we found no evidence of publication bias [Egger’s test (27): 1.79; 95% CI −3.14 to 6.72; *p* > 0.1] (Figure 5). Table 3 shows that the percentage of VAP in the HTV group was lower (−6.1%, 95% CI −27.7 to 20), but this difference was not statistically significant with a low certainty of evidence.

**TABLE 3:** Certainty of the evidence (CoE) through GRADE.

Outcome No. of participants (studies)	Relative effect (95% CI)	Anticipated absolute effects (95% CI)			Certainty
		**Sin (HTV)**	**Con (HTV)**	**Difference**	
396 participants: (5 studies)	OR 0.87 (0.41 to 1.43)	46.9%	40.8% (19.2 to 67.1)	6.1% fewer (27.7 fewer to 20.2 more)	⊕⊕○○ Low

They show a 6.1% lower risk of developing VAP in patients with CSCI using HTV than LTV, but without showing a statistically significant difference and with a low degree of certainty. The risk in the intervention group (and its 95% confidence interval) is based on the assumed risk in the comparison group and the relative effect of the intervention (and its 95% CI). CI, confidence interval; OR, odds ratio; HTV, high tidal volume.

## 4 Discussion

This is the first systematic review and meta-analysis that investigates the effect of MV with HTV compared to LTV in patients with CSCI and its association with pulmonary complications and other undesirable respiratory outcomes. We found that there is no significant difference in the presence of VAP as the main outcome in patients with CSCI on MV if HTV vs. LTV is used (OR 0.48; 95% CI 0.30 to 0.79; *p* < 0.05; *I*^2^: 0%). In turn, there is no significant difference regarding the frequency of pulmonary complications such as ARDS, atelectasis, complications after TQT or delays on initiation of weaning in the HTV group compared to those in the LTV group. However, due to the very limited number of primary studies, the results are inconclusive and should be interpreted with caution.

The main complication in patients with CSCI is VAP, which causes significant morbidity and mortality. Therefore, it is necessary to prevent it and start the weaning the patient with CSCI from the MV as soon as possible; only then can the quality of life be improved and healthcare costs reduced (31–34). Poor lung expansion and secretion clearance lead to the development of pneumonia (35). Therefore, the concept of using HTV in patients with CSCI lies in the fact that the ventilatory pathophysiology in patients with CSCI is different from that in a critically ill patient with lung injury, given that there is less compliance of the respiratory system, composed of the thoracic cage and lung parenchyma. Additionally, the absence of adequate use of abdominal muscles makes achieving adequate TV for these patients more challenging (16, 35, 36). It is reasonable to think that using HTV would lead to controlled overdistension, inducing more outstanding production of surfactant in type II pneumocytes and the alveoli and, therefore, prevent complications such as VAP and atelectasis, among other pulmonary complications (11, 12, 35). Something similar is based on the fact that Gattinoni and Pesenti’s concept of “baby lung” does not intuitively apply to the generally healthy lungs of patients with CSCI. Tetraplegic patients often experience air hunger when LVT ventilation is used, even with normal PaCO_2_, and there is evidence that HTV ventilation may improve weaning success from MV (37, 38).

There are only two RCTs published to date that evaluate MV-dependent CSCI patients and outcomes associated with pulmonary complications (11, 12). Sengupta et al. (12) carried out an RCT, the most currently published, in patients with CSCI where they evaluated the use of HTV compared to LTV and its effect on outcomes such as days to achieve MV release, VAP, atelectasis, and ARDS, enrolling a total of 28 patients for each study group (experimental and control). They found that although there is a higher frequency of VAP in the LTV group compared to HTV (32.14 vs. 10.71%, p: 0.05), there is no statistical significance. The author, when evaluating the role of using HTV with respect to the presence of ARDS, length of hospital stay, and use of vasopressor support, did not find significant differences concerning the LTV group. Only 4 (14%) patients with ARDS were in the HTV group compared to 10 patients (36%) in the LTV group. However, without statistical significance (*p* = 0.14), although there is no data on the PEEP values used in the ARDS groups, higher peak pressure values are shown in HTV vs. LTV (29 vs. 19 mmHg, *p* < 0.01), probably attributed to HTV and PEEP but without more significant evidence of injuries due to barotrauma (pneumothorax, VILI, among others). This demonstrates that the use of HTV is safe. This study is one of the few that assesses mortality as an isolated outcome, where no difference is evident, considering more deaths in the LTV group (9 patients, 37%) compared to the HTV group (5 patients, 18%), but without statistical significance (*p* = 0.22).

On the other hand, Fenton et al. (11) conducted an RCT with 35 tracheotomized patients with CSCI (at level C3-C6) MV dependant, using high TV (HTV, experimental group) at values of up to 20 ml/kg per PBW compared to the control group with 10 ml/kg by PBW of TV (LTV, control group). They found no significant difference in the frequency of VAP in both groups (OR 1.56; *p* > 0.05). Of the total of 7 VAPs found, four were from the LTV group, and three were from the HTV group. They also did not find a significant difference in the presence of ARDS or other complications resulting from barotrauma, concluding that it may be safe to use HTV in patients with CSCI based on those above and on the quantification of forced vital capacity (FVC) of both groups of 1231 ml (HTV group) and 1122 (LTV group), with no significant difference (*p* > 0.05). These findings suggest that using HTV is not harmful and should be evaluated in RCTs with larger population.

In contrast to the two RCTs mentioned, a RCS carried out by Korupolu et al. (7) evaluated the use of HTV as a risk factor for VAP, incorporating 84 patients with CSCI tracheostomized on MV, making up the HTV group with 34 patients and 50 patients in the LTV group. They found that there is a greater risk of VAP with the use of HTV (RR 4.3; 95% CI 1.5 to 1.2; p: 0.01), and although in the general characteristics of the patients, the HTV group has a TV (ml) of 875 vs. 750 in the LTV group, and in the same way the peak pressure (mmHg) 21 vs. 19, respectively; they conclude in the multivariate analysis that the increase of 1 ml in the TV is associated with a lower risk of VAP (RR 1.28; 95% CI 1.1 to 1.6; p: 0.02), including mortality when analyzed as a composite outcome (VAP plus mortality) (OR 1.4; 95% CI 1.1 to 1.8; p: 0.01). It is striking in this study by Korupolu et al. (7) that, on the contrary, in its general characteristics, the group of patients who have HTV has a lower TV compared to the group of patients with LTV. In addition, this study does not carry out sample selection through a probabilistic method, the confounding variables are not necessarily the most appropriate, and the HTV group was considerably older compared to the LTV group. Therefore, the conclusions mentioned above must be interpreted with caution.

The postulated mechanism by which HTV should lead to a lower rate of VAP and other pulmonary complications has yet to be fully understood. However, it is rationally and physiopathological based on the concept that using restrictive TV (LTV or standard, as per our investigation) at 6–8 ml/kg is based on protective lung principles supported by the Acute Respiratory Distress Syndrome (ARDS) Network (13). It is necessary to understand that the evidence supporting lower mortality with low tidal volumes proceed from patients with specific lung pathologies. In contrast, patients with an initial CSCI typically have healthy lungs, suggesting they benefit from using HTV for the aforementioned physiopathological reasons (4, 16, 36).

In addition to the complication due to VAP in patients with CSCI, the presence of atelectasis is a non-negligible situation. The two RCTs that assess this complication found no differences between the presence of atelectasis and the use of tidal volume in HTC or LTV. Fenton et al. (11) report 100% compliance in both groups. Sengupta et al. (12), on the other hand, found a more significant number of atelectasis in the LTV group compared to HTV in 18 patients (64%) compared to 13 patients (46%), respectively, suggesting, again, that the use of HTV can help reduce complications such as atelectasis without entailing problems associated with volutrauma or barotrauma in VM. The same authors also do not report differences in the rate of complications when performing tracheostomy in these groups of patients.

Although there are no significant differences in mortality, in our meta-analysis, when evaluating as a composite outcome (VAP and mortality), excluding Korupolu et al. (7) for behaving as an outlier, we found a reduction in the rate of VAP and mortality in the HTV use group up to 52% less (RR: 0.48; 95% CI 0.30 to 0.79; *I*^2^: 0%) but larger RCT-type studies are needed to demonstrate what is stated in this research.

Our study has numerous strengths. Firstly, we carried out a comprehensive search strategy, which covered six essential databases and clinical trial registers. Secondly, we utilized a rigorous methodology to conduct our review and meta-analysis, which included a thorough quality assessment of studies and a statistical analysis that accounted for heterogeneity. Thirdly, as there was no statistical heterogeneity, it suggests that our findings are dependable and robust. Moreover, the results of individual studies are consistent.

It is important to note that our study has some limitations. First, only a few completed studies have explicitly addressed our PECO/PICO question. Second, the studies included in our meta-analysis were a mix of observational (three) and RCTs (two), with observational studies being more prone to bias than RCTs. Third, while most of the studies used HTV values over 15 ml/K PWB (4, 7, 11, 12, 30), one study used similar but different management points (5). Finally, due to the limited number of studies available, we had to analyze both observational and RCTs, considering the moderate risk of bias found in the reported observational studies.

## 5 Conclusion

Our study suggests that there is no significant difference in the development of VAP as a complication when using HTV compared to LTV in patients with CSCI in MV, nor are there differences in the presentation of other pulmonary complications such as ARDS, atelectasis, and onset of MV weaning. There is also no evidence that there are more complications associated with volutrauma in the HTV group, indicating that this strategy could be safe. In the composite outcome, when evaluating both VAP and mortality, the results suggest a lower rate of VAP and mortality by up to 52% after excluding outliers. The conclusions above have a low level of evidence. RCTs are necessary to demonstrate the effectiveness of using HTV in patients with CSCI or to rule it out definitively and to be able to couple this evidence into management guidelines.

## Data availability statement

The raw data supporting the conclusions of this article will be made available by the authors, without undue reservation.

## Author contributions

EM-R: Conceptualization, Data curation, Formal Analysis, Funding acquisition, Investigation, Project administration, Software, Supervision, Writing – original draft. GV-T: Conceptualization, Data curation, Formal Analysis, Funding acquisition, Investigation, Project administration, Software, Supervision, Writing – original draft, Methodology, Resources, Validation, Validation, Writing – review and editing. CQ-C: Data curation, Formal Analysis, Methodology, Resources, Visualization, Writing – original draft. MC: Funding acquisition, Investigation, Project administration, Resources, Software, Validation, Visualization, Writing – original draft. JC-C: Formal Analysis, Funding acquisition, Investigation, Methodology, Resources, Writing – review and editing. JP-P: Conceptualization, Writing – original draft.
