# Supplementary material for: High vs. low tidal volume and pulmonary complications in patients with cervical spinal cord injury on mechanical ventilation: systematic review
Source: Front Med (Lausanne). 2024 Mar 1;11:1362318. doi: 10.3389/fmed.2024.1362318 (PMC10940362; doi:10.3389/fmed.2024.1362318)
Supplement: Supplementary file 1 [file Table_1.docx]

Table S1A- Search strategy

| 1. MEDLINE VIA PUBMED | | Results | Date |
| --- | --- | --- | --- |
| #1 | **(vertebro-medullary injuries) OR (vertebro-medullary trauma)) OR (Spinal Cord Injuries)) OR (Spinal Cord Trauma)) OR (Trauma, Spinal Cord)) OR (Traumatic Myelopathy)) OR (Transection, Spinal Cord)) OR (Contusion, Spinal Cord)** | 70,578 | 30/August/2023 |
| #2 | **(mechanical ventilation weaning) OR (weaning)) OR (invasive mechanical ventilation)) OR (Artificial Respiration)) OR (Ventilator Weaning)) OR (Tidal Volumes)** | 156,121 |  |
| #3 | #1 AND #2 | 574 |  |

| 2. EMBASE | | Results | Date |
| --- | --- | --- | --- |
| #1 | ('spinal cord injury'/exp OR 'spinal cord injury' OR 'spinal cord'/exp OR 'spinal cord' OR 'cervical spinal cord injury'/exp OR 'cervical spinal cord injury' | 320 344 | 30/August/2023 |
| #2 | ('artificial ventilation'/exp OR 'artificial ventilation' OR 'invasive ventilation'/exp OR 'invasive ventilation' OR 'tidal volume'/exp OR 'tidal volume') AND ('ventilator weaning'/exp OR 'ventilator weaning') OR 'tidal volume' | 37184 |  |
| #3 | #1 AND #2 | 440 |  |

| 3. WEB OF SCIENCE | | Results | Date |
| --- | --- | --- | --- |
| #1 | **( "spinal cord injury" OR "cervical spinal cord injury" OR "'cervical spinal cord injury" )** (All Fields) | [56 781](https://webofscience.upao.elogim.com/wos/woscc/summary/a71cabe6-7984-45f3-9868-6a11dbe95210-73415b19/relevance/1) | 30/August/2023 |
| #2 | **( "ventilator weaning" OR "'artificial ventilation" OR "'invasive ventilation" OR "'tidal volume" OR "'invasive ventilation" OR "'artificial ventilation" )** (All Fields) | [20 619](https://webofscience.upao.elogim.com/wos/woscc/summary/99782b7a-41a1-4807-b72a-33dc0ecad780-73415d04/relevance/1) |  |
| #3 | #1 AND #2 | 176 |  |

| 4. SCOPUS | | Results | Date |
| --- | --- | --- | --- |
| #1 | TITLE-ABS-KEY ( "spinal cord injury" OR "cervical spinal cord injury" OR "'cervical spinal cord injury" ) | 77 734 | 30/August/2023 |
| #2 | TITLE-ABS-KEY ( "ventilator weaning" OR "'artificial ventilation" OR "'invasive ventilation" OR "'tidal volume" OR "'invasive ventilation" OR "'artificial ventilation" ) | 171 522 |  |
| #3 | ( TITLE-ABS-KEY ( ventilator AND associated AND pneumonia ) ) | 14 712 |  |
| #4 | (#1 AND #2) AND #3 | 50 |  |

| 5. SCIENCE DIRECT | | Results | Date |
| --- | --- | --- | --- |
| #1 | TITLE-ABS-KEY ( "spinal cord injury" OR "cervical spinal cord injury" OR "'cervical spinal cord injury" ) | 13 968 | 30/August/2023 |
| #2 | TITLE-ABS-KEY ( "ventilator weaning" OR "'artificial ventilation" OR "'invasive ventilation" OR "'tidal volume" OR "'invasive ventilation" OR "'artificial ventilation" ) | 6 024 |  |
| #3 | ( TITLE-ABS-KEY ( ventilator AND associated AND pneumonia ) ) | 41 |  |

Table S1B. Excluded studies for meta-analysis and the reason for their exclusion

| Study | Reason for exclusion |
| --- | --- |
| Wong S, Shem K, Crew J. Specialized respiratory management for acute cervical spinal cord injury: A retrospective analysis. Topics in Spinal Cord Injury Rehabilitation. 2012;18(4):283-290. doi:10.1310/sci1804-283 | No adecuate data. |
